# Active aging awareness and well-being among older adults in Portugal

**DOI:** 10.3389/fpubh.2023.1149731

**Published:** 2023-04-12

**Authors:** Andreia Costa, Joana Henriques, Violeta Alarcão, Adriana Henriques, Teresa Madeira, Ana Virgolino, Joana Sousa, Rodrigo Feteira-Santos, Miguel Arriaga, Jorge Rocha, Paulo Nogueira

**Affiliations:** ^1^Nursing Research, Innovation and Development Centre of Lisbon (CIDNUR), Nursing School of Lisbon, Lisbon, Portugal; ^2^Instituto de Saúde Ambiental (ISAMB), Faculdade de Medicina, Universidade de Lisboa, Lisbon, Portugal; ^3^Laboratório para a Sustentabilidade do Uso da Terra e dos Serviços dos Ecossistemas – TERRA, Lisbon, Portugal; ^4^Católica Research Centre for Psychological-Family and Social Wellbeing (CRC-W), Faculdade de Ciências Humanas, Universidade Católica Portuguesa, Lisbon, Portugal; ^5^Escola Nacional de Saúde Pública, ENSP, Centro de Investigação em Saúde Pública, CISP, Comprehensive Health Research Center, CHRC, Universidade NOVA de Lisboa, Lisbon, Portugal; ^6^Centro de Investigação e Estudos de Sociologia (CIES-Iscte), Instituto Universitário de Lisboa (Iscte), Lisbon, Portugal; ^7^Instituto de Geografia e Ordenamento do Território da Universidade de Lisboa (IGOT-ULisboa), Lisbon, Portugal

**Keywords:** active and healthy aging, well-being, older adults, social inequalities, Portugal

## Abstract

**Objective:**

This study aims to assess the active aging awareness of older adults in mainland Portugal and their levels of overall well-being and to identify social and health-related factors.

**Methods:**

A cross-sectional study was conducted with a representative sample of 613 older adults, aged 65 or older, who participated in the PROKnos – Knowing Social Prescribing needs of the elderly study in Portugal. The questionnaire consisted of the Active Ageing Awareness Questionnaire and the World Health Organization – Five Well-Being Index, as well as sociodemographic, economic, and health status questions. Correlation coefficients, t-tests for independent samples, and one-way ANOVA were used to explore potential associations between variables.

**Results:**

The active aging awareness levels were significantly higher for women (*p* = 0.031), and those who were younger (*p* = 0.011), more educated (*p* < 0.001), had a better financial situation (*p* < 0.001), and had better health (*p* < 0.001). The same pattern was found for well-being, except in relation to gender, as men had higher levels (*p* = 0.016). These variables were found to be correlated.

**Discussion:**

Even though active aging is an important strategy to implement, it is indispensable to consider the perceptions and conditions that need to be in place before that. This study reveals that several social and health-related factors are associated with well-being and active aging awareness, as well as the differences between groups that exist in mainland Portugal in relation to that. This emphasizes how vital it is to address social inequalities in active aging efforts, which are not necessarily uncovered when only considering actual active aging measures.

## Introduction

There have been significant increases in life expectancy at birth over the last few decades in most countries. As much as this means that people generally enjoy longer lives, it also conveys that the population is aging rapidly (1, 2). The proportion of people aged 60 or over has been growing faster than any other age group worldwide, a trend that was reported at the beginning of the century by the World Health Organization (WHO) and is predicted to keep increasing exponentially (3). In 2019, 65-year-old individuals could expect to live approximately an additional 20 years on average in Organization for Economic Co-operation and Development (OECD) countries (4). Data from 2021 shows that 20.8% of the population in the European Union (EU) was 65 or over, and there are estimates that people in this age group will account for 31.3% of the population by 2100 (5). The median age of the EU population has been increasing steadily and is expected to continue rising at the same rate for the next 20 years (1).

The marked aging of the population represents one of the biggest challenges currently faced due to the significant impact it brings to societies, spanning from economic and healthcare sustainability to intergenerational social cohesion, along with the older adults’ well-being and quality of life (2). Despite the important strides in increasing life expectancy, not all of the additional years are lived in good health (e.g., from the added 20 years previously mentioned, only approximately 10 would be considered healthy life years) (4). The longer individuals live, the higher the chance of suffering from chronic illnesses or disabilities, often presenting comorbidities. In 2018, 7.6% of EU workers between the ages of 18 and 64 reduced their working time or took considerable work leaves to take care of sick or older relatives with disabilities. The reason why this is especially troublesome is twofold: the number of people who potentially need long-term care is likely to keep escalating in the EU; and relying on the help of informal carers is no longer as viable, since families are having fewer children, living farther apart, and more women are employed (2).

Arguably, the most debated solution to this issue has been active aging, which can be defined as the process of optimizing opportunities for health, participation, and security to enhance the quality of life as people age (3). Active aging aims to promote people’s conscious participation in society, in their community, and their physical and mental well-being throughout the life course, including their older years. To achieve this, it is necessary to consider older adults’ needs, interests, and, more importantly, capabilities. Active aging is promoted through continued participation in many aspects of life (e.g., cultural affairs), without hindering those individuals who are ill, have disabilities, or are limited by mobility issues. Given older adults’ heterogeneity as a group, it is crucial to broaden the notion of what it means to be active (6). Active aging goes, therefore, well beyond the ability to be physically active or remain in the workforce after retirement, focusing instead on empowering people to take an active stance in extending their healthy life expectancy and ensuring autonomy and independence as they age (3).

In light of this perspective, the unavoidable aging phenomenon becomes one of new opportunities and solutions to foster social fairness, rather than a problem to overcome (2). It switches the narrative from older people being passive targets to acknowledging that people have the right to equal opportunities as they age (3). As a result of medical improvements and health promotion efforts, most older adults could maintain good health for longer, enabling them to choose how to spend their time. This new reality challenges previous perceptions of aging that were associated with decline, shining a light on the important contribution of older adults to their families, communities, and economies by being an added resource (2, 7).

In Portugal, demographic aging has been increasing in a very expressive way, with the country presenting one of the lowest rates of young people (13.4%) and one of the highest shares of older people (22.4%) in the EU in 2021 (5). Looking at particular metrics, the aging index shows that there are 182 older people for every 100 young people, an increase from 128 in just a decade (8, 9). In that same period, the average age of the population in Portugal increased by 3.1 years, standing at 45.4 and surpassing the European average (44.1) (8). However, the results from the Active Ageing Index (AAI) project indicate that Portugal’s score was still 2.2 points lower than the EU average in 2018, being particularly low in the ‘participation in society category’ (10). The country has been making important strides, namely with the nomination of a working group to develop the National Strategy for Active and Healthy Aging (ENEAS), but there is still room for improvement (11, 12).

Aging is experienced differently by everyone because sociodemographic, economic, and professional aspects play an important part in health and perceived well-being, to a greater degree in older age (13). For example, some argue that most active aging policies are “gender blind” because they do not take into account the very different challenges that men and women face during their life course, which has severe consequences in the way they age and their levels of well-being (14). In order to ensure every person is included in healthy and active aging efforts, policies must reflect specific needs. This principle is highlighted in the plan to improve functional ability in older adults in Portugal, through meaningful engagement with older people and the importance of delivering person-centered and integrated care in these age groups (15). Some studies have been developed in Portugal to assist in the assessment of active aging and explore priorities that support adequate policies (16–18), but this might not be enough to get a clear understanding of people’s true needs and perceptions about active aging. Based on our understanding, older adults’ knowledge about active aging has never been explored in Portugal with older adults, even though it might provide important information to move active aging policies and action plans forward. For that reason, this study aims to assess the active aging awareness of older adults in mainland Portugal and their level of overall well-being and to identify social and health-related factors that are associated with those variables.

## Materials and methods

### Study design and ethics

This study draws on data from the PROKnos – Knowing Social Prescribing needs of the elderly study that aimed to better understand the needs of older adults in Portugal in relation to social prescribing as a strategy to support healthy aging.

A cross-sectional study was conducted between September and October 2022 in mainland Portugal, with a representative sample of older adults. Participants were randomly selected from a list of landline and cell phone numbers, also randomly generated by a specialized polling center, that had been used in a previous study. The inclusion criteria were as follows: 65 years-old or older adults living in mainland Portugal.

Data were collected through a telephone survey, with an average duration of 37 min, conducted by trained interviewers using a computer-assisted structured telephone interview system. Individuals agreed to participate by giving oral consent during the phone call, after being informed about the study’s aim and that their participation would be completely voluntary and anonymous. In total, 1916 older adults were contacted but 1303 were excluded for not answering the phone after three separate attempts (*N* = 159), not meeting the inclusion criteria (e.g., younger than 65; *N* = 924), and refusing to participate (*N* = 220). This study’s response rate was 74%, which resulted in 613 valid questionnaires. The maximum margin of error associated with a random sample of 613 respondents is 4% with a confidence level of 95%.

The study was approved by the Ethics Committee of the Centro Académico de Medicina de Lisboa (Process number 193/22) and was implemented in compliance with the ethical principles set out in the Declaration of Helsinki. Compliance with the General Data Protection Regulation (GDPR) and national legislation has been guaranteed by securing all data on restricted computer systems, to which only the researchers are granted access, and by linking each file to a random code that cannot be used to identify the subjects.

### Measures

In this study, two dimensions of healthy aging were considered: active aging awareness and overall well-being. Participants were also asked about their sociodemographic characteristics (e.g., education level), economic situation (e.g., “how do you rate the current financial situation of your household”), and general health status (e.g., “how would you rate your overall health”).

The Active Ageing Awareness Questionnaire (AAAQ) consists of two stand-alone questions and 14 items and was used to assess active aging awareness (19). The two stand-alone questions can be analyzed separately. The first question asks whether the participants have heard of the term ‘active aging’. The second question is open-ended, and the participants are asked to give their opinion about factors that may help them age actively. Participants’ responses to the 14 items in the AAAQ were scored using a 4-point Likert scale and were summed to form a score ranging from 14 to 56. The score was then converted to a 0–100 scale, following the conversion method provided by the authors. The AAAQ showed excellent internal consistency in this study (*α* = 0.91).

The World Health Organization - Five Well-Being Index (WHO-5) is a short self-reported measure to study overall well-being (20). It consists of five statements, which respondents rate according to a 6-point Likert scale ranging from 5 - All of the time to 0 – At no time, in relation to the past 2 weeks. The total raw score, ranging from 0 to 25, is multiplied by 4 to give the final score, with 0 representing the worst and 100 representing the best imaginable well-being. Research (21) has shown its adequate validity as an outcome measure in a wide range of fields. In this study, the WHO-5 showed good internal consistency (*α* = 0.85).

### Statistical analysis

Responses were analyzed using IBM SPSS 27 (IBM Corp. Released 2020. IBM SPSS Statistics for Windows, Version 27.0. Armonk, NY: IBM Corp). Categorical and numerical variables were characterized using frequencies and descriptive statistics. Bivariate analyses were performed, namely correlation coefficients, t-tests for independent samples, and one-way ANOVA to explore potential associations between variables. The significance level considered was *p* = 0.05.

## Results

Six hundred and thirteen older adults participated in this study, of which 52.2% were male, and whose ages ranged from 65 to 93 (*M* = 72.8; *SD* = 5.80). The majority were married (63.5%), lived with at least one person (52.4%), were retired (88.4%), and were of Portuguese nationality (94.3%). Almost half of the participants considered their household income sufficient for their needs (41.9%). However, a considerable portion of the sample (29.6%) found it difficult or very difficult to live with their income. As for their educational background, 27.6% had concluded the fourth grade (primary school). Most participants lived in the Lisbon Metropolitan Area (40.1%). For more sociodemographic information, please see Table 1.

**Table 1:** Sociodemographic characteristics.

	*N*	%
Sex	613	–
Male	320	52.20
Female	293	47.80
Age, *years (M = 72.84; SD = 5.79)*	613	–
65–74	405	66.1
75–84	183	29.9
>84	25	4.1
Civil status	613	–
Single	29	4.70
Non-marital partnership	6	1.00
Married	389	63.5
Separated	6	1.00
Divorced	75	12.20
Widowed	108	17.6
Educational level	613	–
Illiterate	1	0.20
Able to read and write (no schooling)	2	0.30
First cycle of basic education (4th grade, primary school)	169	27.60
Second cycle of basic education (6th grade)	38	6.20
Third cycle of basic education (9th grade)	83	13.50
Secondary education (12th grade)	131	21.4
Undergraduate/Medical degree	33	5.40
Bachelor’s degree	131	21.40
Master’s degree	13	2.10
PhD	12	2.00
Employment situation	613	–
Full-time	30	4.90
Part-time	8	1.30
Unemployed	4	0.70
Retired	542	88.40
Never worked outside the home	11	1.80
Permanently unable to work	9	1.50
Informal caregiver	2	0.30
Other	7	1.20
Household’s income situation *(M = 3.08; SD = 0.97)*	608	–
Very comfortable	17	2.8
Comfortable	156	25.4
Sufficient for the household’s needs	255	41.6
Difficult	122	19.9
Very difficult	58	9.5

When queried about health-related variables (Table 2), almost half (49.8%) considered their health condition acceptable, and 53.2% claimed to have no illness or disability. Nearly all the participants (95.3%) were registered in a primary health care unit, with 68.8% having had at most one appointment with a doctor or nurse in the last 6 months. Overall, the health care provided was rated as reasonable (*M* = 3.50; SD = 0.99).

**Table 2:** Health-related characteristics.

	*N*	%
Overall health rating (*M* = 2.84; *SD* = 0.87)	613	–
Very good	39	6.4
Good	155	25.3
Acceptable	305	49.8
Bad	94	15.3
Very bad	20	3.3
Suffering from an illness or disability	613	–
Yes	287	46.80
No	326	53.20
Enrolled in a primary healthcare unit	613	-
Yes	584	95.3
No	29	4.7
Number of visits in the last 6 months	584	–
0–1	401	68.80
>2	183	31.20
Rating of the care provided at the healthcare unit (*M* = 3.50; *SD* = 0.99)	529	-
Very good	85	16.10
Good	186	35.20
Acceptable	184	34.80
Bad	55	10.40
Very bad	19	3.60

**Figure 1:**
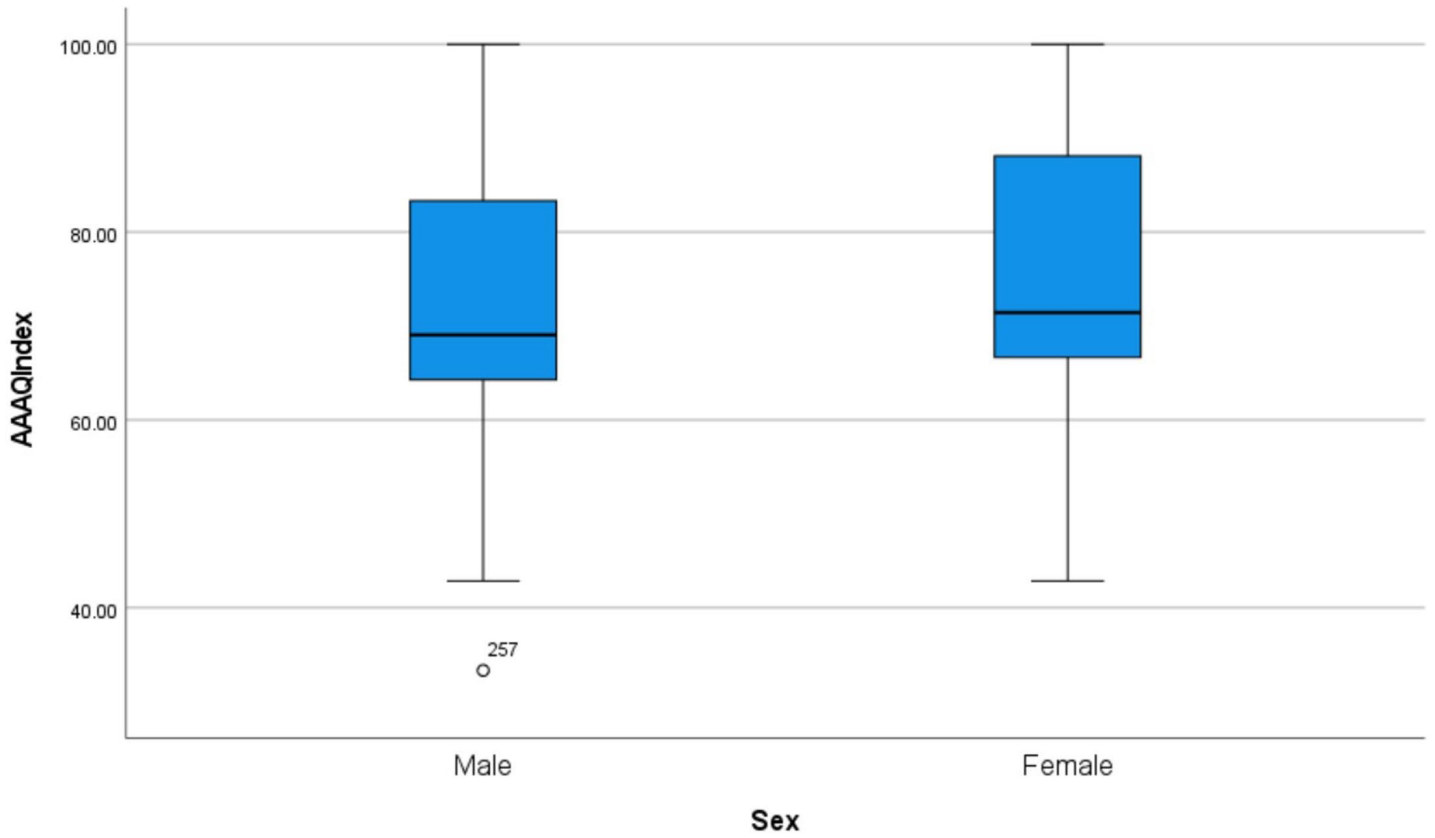
Active aging awareness scores by sex.

**Figure 2:**
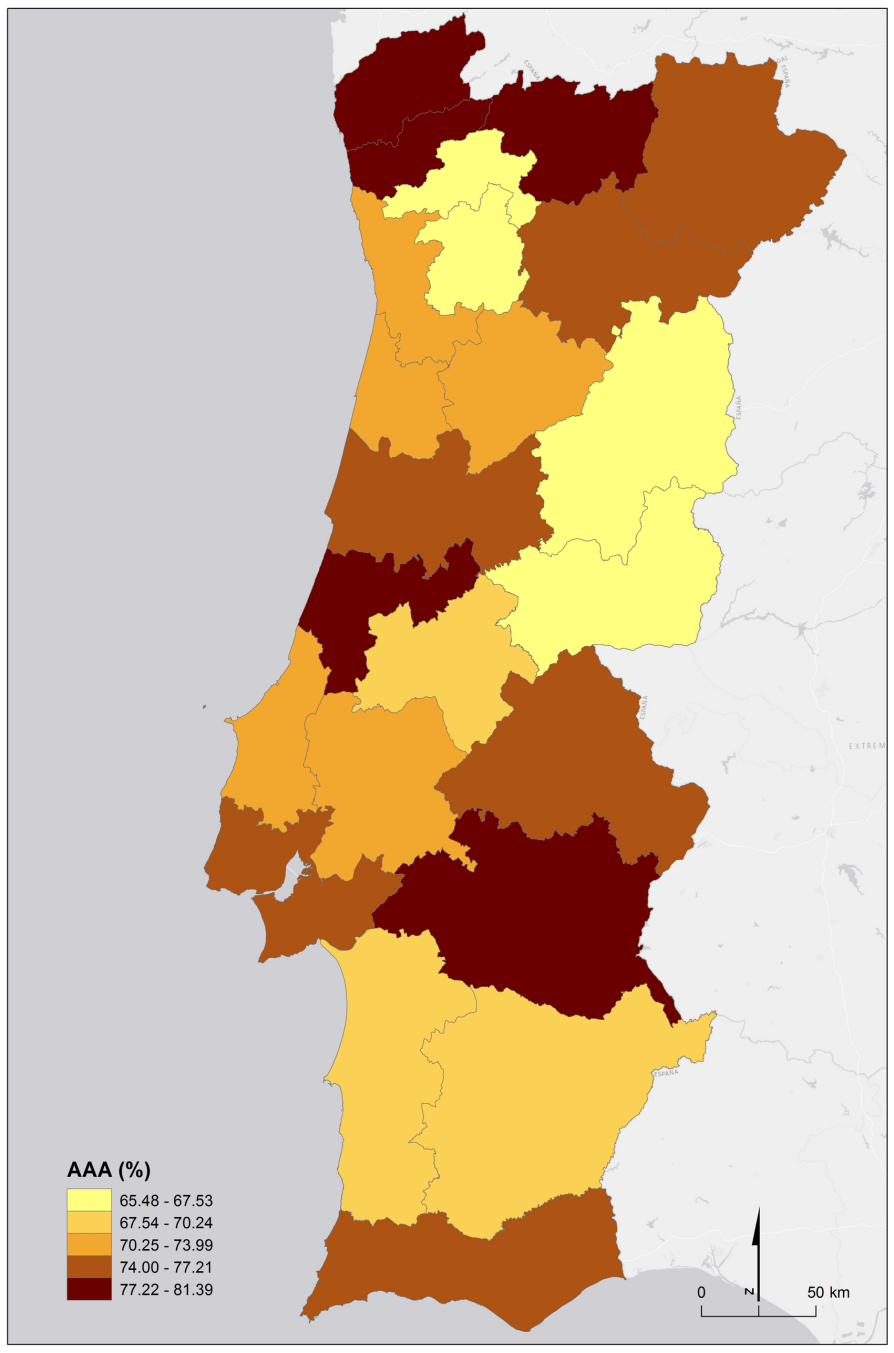
Active aging awareness scores by NUTS III (mean).

**Figure 3:**
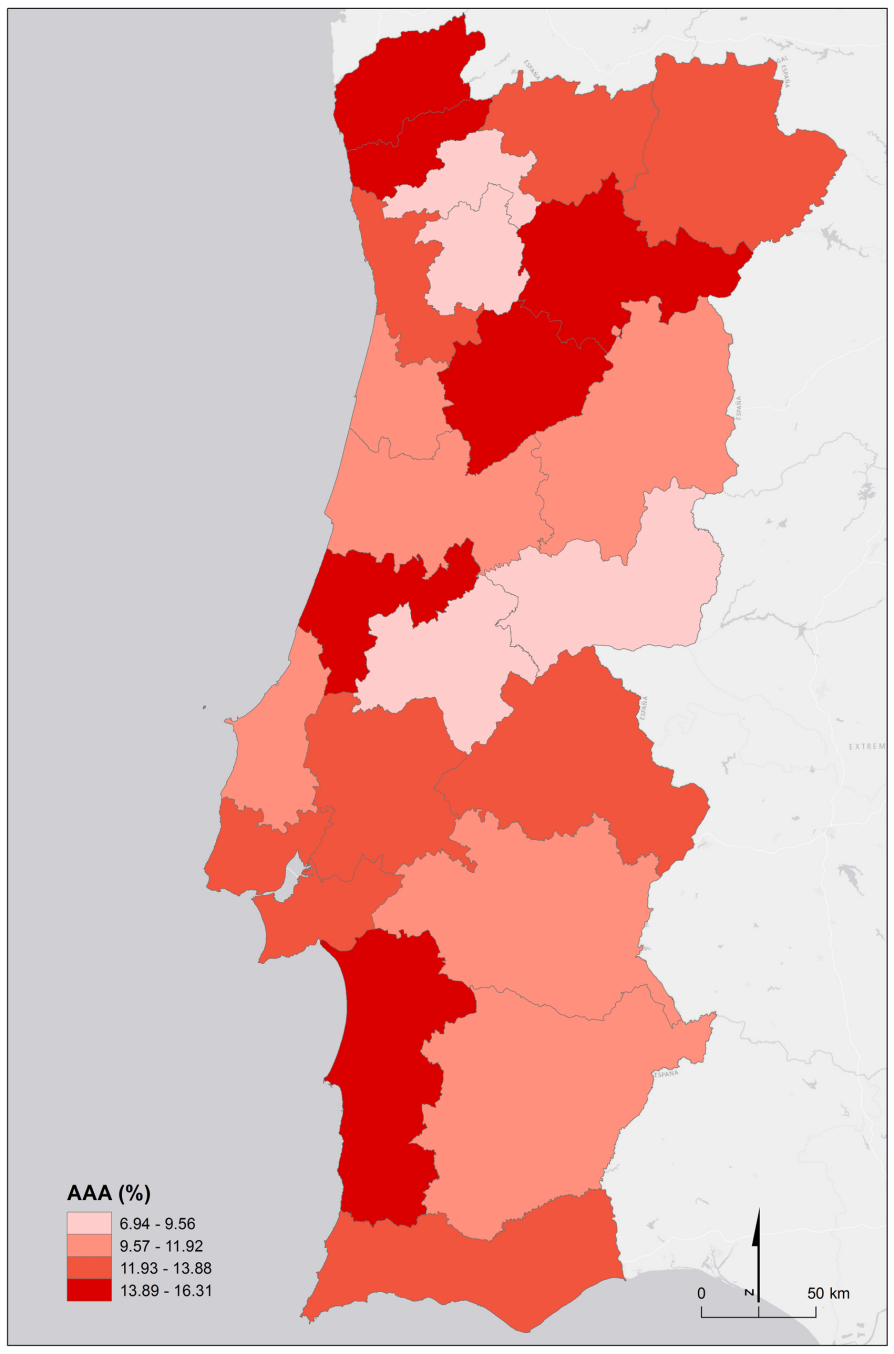
Active aging awareness acores by NUTS III (standard deviation).

Concerning active aging, 72.9% of the sample reported having heard about this subject before. Most participants were also able to name factors that are important for aging actively, such as staying physically active, pursuing hobbies, and spending time with friends and family regularly. When presented with a list of factors that may promote active aging, participants agreed the most on the importance of having a healthy mind, eating healthy food, continuing to learn new things, and having a home. On the other hand, some disagreed with the idea that not consuming alcoholic beverages and being free of chronic diseases were conditioning factors for active aging. Results from the AAAQ indicate that the active aging awareness’ mean score was 74.73 (*SD* = 12.95), with women showing higher awareness levels than men [*M* = 75.91; *SD* = 13.36; *M* = 73.65 vs. *SD* = 12.49, respectively; *t*_(611)_ = −2.165, *p* = 0.031]; please see Figure 1. This awareness decreased as age increased [*F*_(3)_ = 3.723; *p* = 0.011], but it increased with educational level [*F*_(9)_ = 3.604; *p* < 0.001] and with a better financial situation [*F*_(4)_ = 4.559; *p* < 0.001]. Likewise, those who perceived having a better health status [*F*_(4)_ = 6.374; *p* < 0.001], had no illnesses or disabilities [*t*_(608)_ = −3.261; *p* = 0.001], and had, at most, one appointment at the primary healthcare unit in the past 6 months [*t*_(582)_ = 2.041; *p* = 0.042] had a higher index score. Participants in the Alto Minho region presented the highest active aging awareness index (*M* = 81.39; *SD* = 15.93) and the ones in the Ave region presented the lowest (*M* = 65.48; *SD* = 9.47), although the differences between NUTS III were not significant (*p* = 0.073); please see Figures 2, 3. Regarding current work status, people who were still working part-time showed the highest active aging awareness levels, albeit not significant (*p* = 0.227).

**Figure 4:**
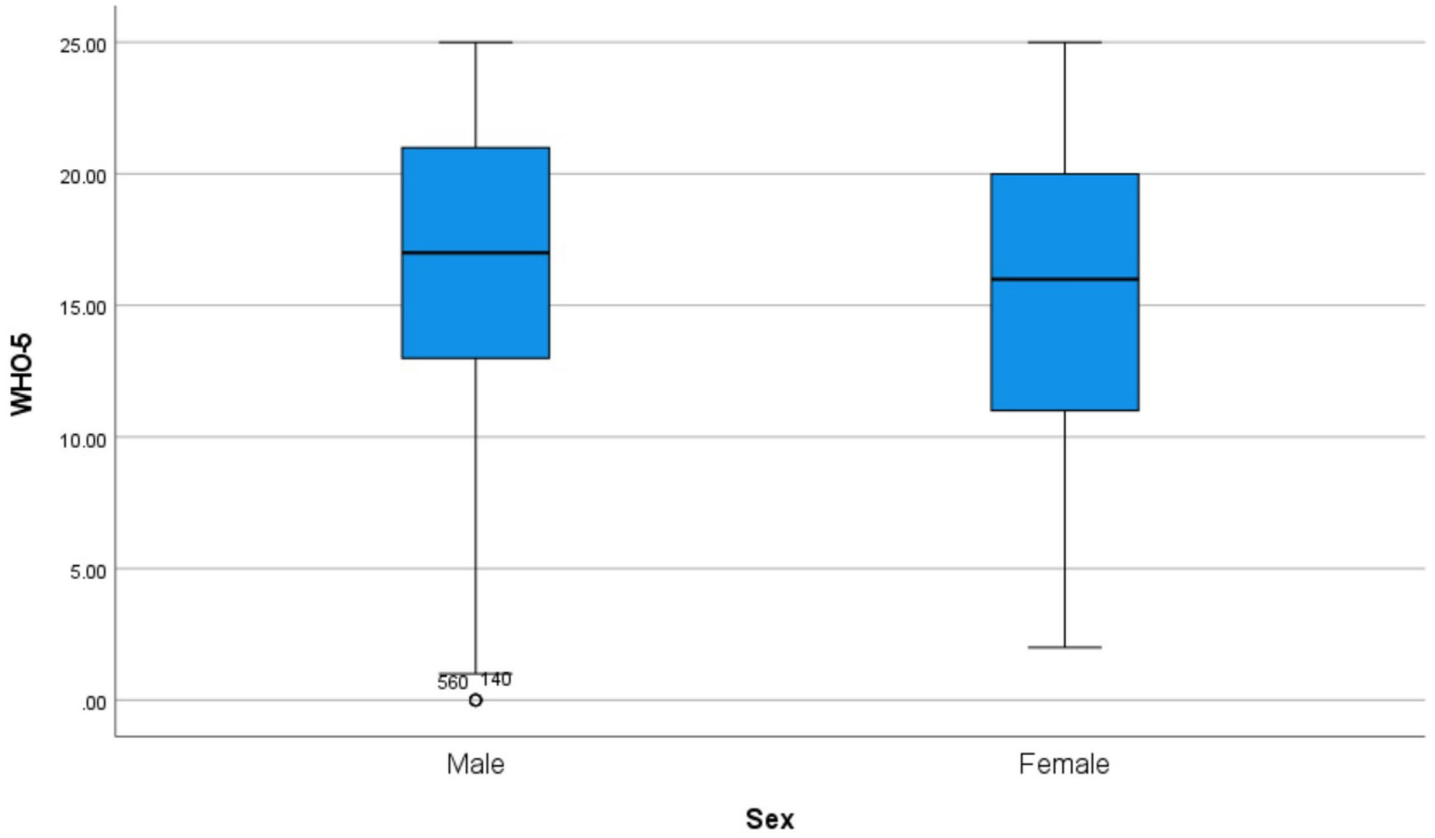
Well-being index scores by sex.

**Figure 5:**
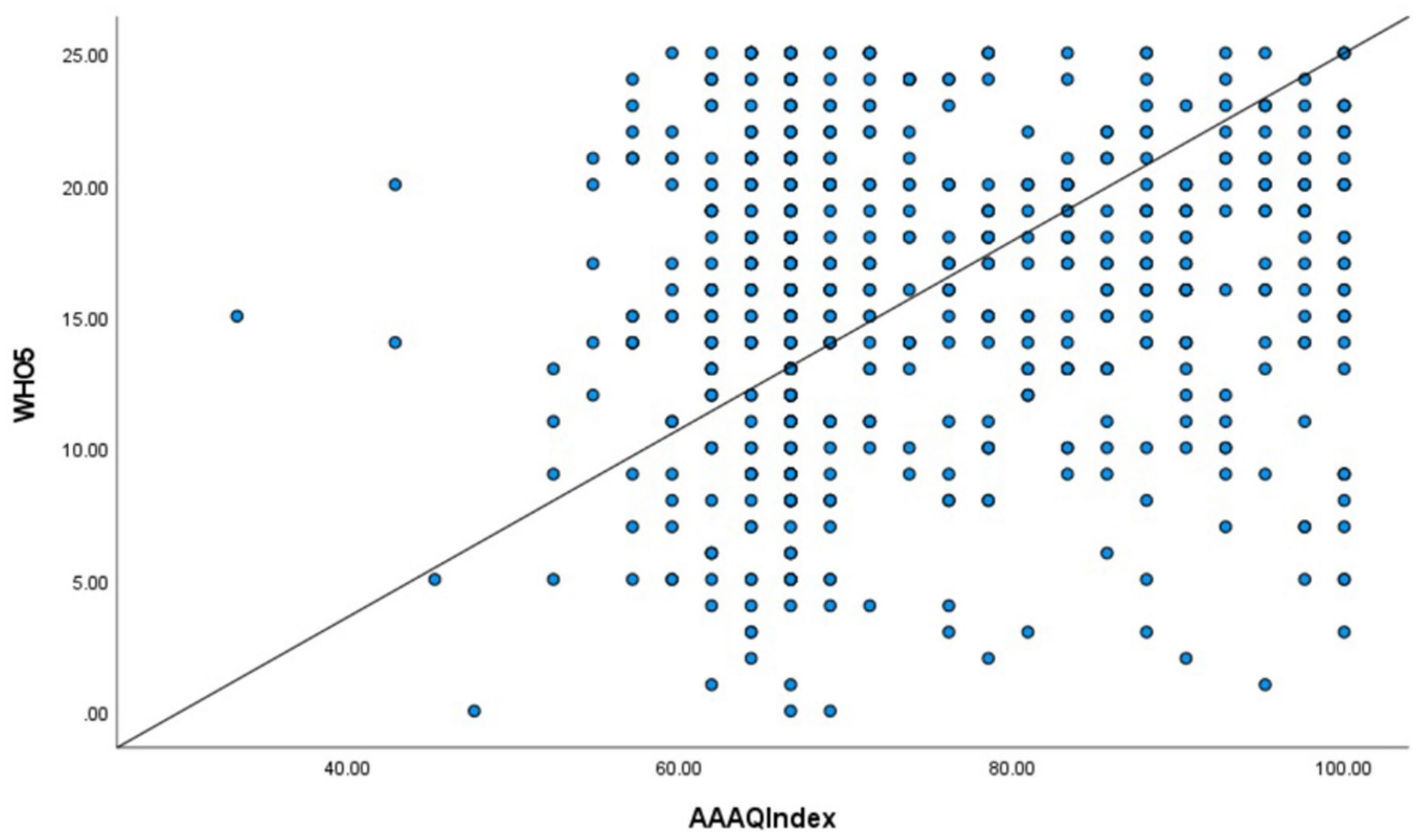
Correlation between active aging awareness and well-being scores.

In terms of well-being, the mean score assessed by the WHO-5 was 63.60 (SD = 5.82), in this case with men having a slightly higher score than women [*M* = 65.80; *SD* = 23.18 vs. *M* = 61.27; *SD* = 23.17, respectively; *t*_(611)_ = 2.418; *p* = 0.016]; please see Figure 4. The highest well-being levels were seen in the Baixo Alentejo region (*M* = 77.00; SD = 17.40), and the lowest in the Tâmega and Sousa regions (*M* = 45.33; SD = 20.98), but once again, the differences between regions were not significant (*p* = 0.600). Similarly to what was observed for active aging, participants who had higher educational level [*F*_(9)_ = 3.165; *p* < 0.001], a better financial situation [*F*_(4)_ = 14.286; *p* < 0.001], a better-perceived health status [*F*_(4)_ = 41.057; *p* < 0.001] and had no illnesses or disabilities [*t*_(611)_ = −5.197; *p* < 0.001] showed higher levels of well-being. This was likewise seen for those who had, at most, one appointment in the healthcare center in the past 6 months [*t*_(582)_ = 3.196; *p* = 0.001]. Looking into work status, people who worked part-time showed the highest active aging awareness [*F*_(9)_ = 2.618; *p* = 0.006]. Moreover, a weak and positive association was found between well-being and active aging awareness [*r*_(611)_ = 0.12, *p* = 0.004]; see Figure 5.

## Discussion

The world’s population is aging rapidly, and Portugal is no exception. Yet, as much as life expectancy has been increasing, this has not been matched by a proportional increase in healthy life expectancy (4). The most recent AAI data showed that more investment is needed in Portugal when it comes to active aging (22), and this can only be accomplished if older adults are engaged and included in this effort. The cross-sectional study presented here was conducted in mainland Portugal to assess older adults’ overall well-being and active aging awareness and to identify social and health-related factors.

Our results show that most of the participants were acquainted with the concept of active aging and were able to provide/select examples of contributing factors. The average AAAQ score was satisfactory, but it was possible to see significant differences between men’s and women’s active aging awareness, a finding that might shed light on Steinmayr and colleagues’ findings (2020). These authors reported that the biggest gender differences in active aging scores were found in Mediterranean countries, including Portugal, and that active aging scores were higher for men (13). The present study, on the other hand, showed that women had higher levels of awareness about the topic, potentially highlighting the gap between reality and their desired situation. The greatest gender differences were noticeable in relation to the importance of taking care of one’s health (not drinking alcohol, having a healthy mind), staying active (doing volunteer work, having hobbies), and utilitarian issues (saving money for retirement, having a home). Data suggests that although women live longer, they tend to experience more disabilities, most of which occur during old age. This could partly explain why women in this sample placed more importance on their health (13, 23). Additionally, women often take on the role of family caregivers, even as they grow older. Faced with this reality, this may explain why they would value having the opportunity to have hobbies or do volunteer work, as it would mean they are able to choose how to enjoy their free time (23). Lastly, there are substantial gender inequalities in older adults’ active aging scores related to employment and income (fewer women from this cohort were employed and educated, earning less money than men and being more dependent on their husbands). For that reason, older women face a higher risk of poverty due to big differences in income (pensions), which might justify why they placed more importance on the ability to have savings and a home (a place to live) (13, 23). Since these factors are of special relevance for women, they could help explain why their active aging awareness level is higher.

At the same time, older adults with better health (i.e., perceived health condition, absence of illness and/or disabilities, and fewer primary healthcare appointments), a better financial situation, and more years of education showed higher levels of active aging awareness. On the other hand, those levels diminished as age increased. These results are in line with the literature, which has shown that better living and health conditions are related to higher active aging, whereas older age is not (24, 25).

Socioeconomic factors, and, particularly, educational level, are positively and strongly related to people’s health, possibly because those who have more schooling usually have more resources and financial stability (26), which provides them with a better chance to adapt to changes brought on by aging and better access to healthcare, resulting in better health and quality of life in advanced age. In parallel, educational levels have been found to be associated with health literacy (27, 28), which might account for people with more education being aware of the importance of aging actively and which factors can contribute to it. It seems plausible to suppose that having awareness about active aging is one of the prerequisites for achieving it, which could explain why the educational level was also associated with active aging awareness in this sample. Despite not having a significant association, it was interesting to find that older adults who were still working part-time had higher active aging awareness, perhaps highlighting the importance of their continued participation and active contribution as suggested by the WHO (3).

As for well-being, the general mean score assessed by the WHO-5 was fair and significantly lower for women in all subdimensions of the scale. This is in agreement with what has been reported in the literature about well-being, i.e., women are more likely to rank themselves lower than men on these types of measures, and this gap increases for older people, mostly because of the social, income, and health-related issues reported above (13, 23). In addition, women also report feeling more worried, anxious, and stressed, which could be linked to their poorer scores in the “calm” and “rested” categories (23).

Seniors who have the opportunity for personal growth and development in old age experience higher levels of psychological well-being and life satisfaction (29). Assuming the same premise, people with higher educational levels and a better financial situation should have more resources and control over their life, even in older years. This could explain why individuals who scored higher in those dimensions in our study also showed higher well-being. In turn, higher well-being has been associated with better health (30), which was also found in our study. Although more wide-ranging, active aging focuses on the idea that older adults should be able to participate in all aspects of society, including paid roles, if they are capable and willing. By remaining engaged and active, older adults continue to learn, have a sense of purpose, are less isolated, and can potentially avoid old age poverty, which would then have a positive impact on their health and well-being (2). Our data showed that working part-time was significantly associated with well-being, supporting those ideas.

The mean distribution for both the WHO-5 index and the AAAQ (per NUTS III) shows a clear geographical heterogeneity throughout the country. The most contrasting levels were found between Baixo Alentejo (highest) and Tâmega e Sousa (lowest) for well-being, and Alto Minho (highest) and Ave (lowest) for active aging awareness. We were not able to find possible reasons in the literature to explain these spatial differences, which are most likely multifactorial in nature. Understanding these differences grants further investigation in future studies.

### Strengths and limitations

This study has several strengths and limitations. For the former, it is important to note the up-to-date quality of the data collected with a representative sample in mainland Portugal. This work adds to the body of evidence in active aging, contributing with findings on older people’s active aging awareness, going beyond the measurement of active aging itself and serving as its complement. For the latter, it would have been helpful to have collected active aging levels to compare participants’ perceptions and their actual active aging situation to explore the discrepancy elicited by the most recent AAI results and the awareness data we gathered. Furthermore, including a qualitative measure like individual interviews could have allowed us to gain a deeper understanding of this gap.

## Conclusion

This study’s results reveal a myriad of social and health-related factors associated with well-being and active aging awareness, having potential implications for actual active aging levels and for the work that must be undertaken to intervene in this context. Our results drew attention to the existing relevant differences across mainland Portugal, such as between men and women or people with different socioeconomic situations. This emphasizes how vital it is to address social inequalities in active aging efforts, respecting the diversity of older adults, their differing needs, and socioeconomic conditions. Further research is necessary to explore these aspects more thoroughly and to ensure everyone is covered by active and healthy aging policies. Such policies could, for example, be facilitated by guaranteeing women have as much access to free time activities (e.g., community clubs) as men do, or by having free services (e.g., counseling) available for people with lower income.

## Data availability statement

The raw data supporting the conclusions of this article will be made available by the authors, without undue reservation.

## Ethics statement

The studies involving human participants were reviewed and approved by Ethics committee of Centro Académico de Medicina de Lisboa. The patients/participants provided their written informed consent to participate in this study.

## Author contributions

AC, AH, VA, MA, and PN: conceptualization, methodology, and validation. AC, PN, JR, and TM: formal analysis. AC and JH: writing—original draft preparation. AC, AH, VA, MA, PN, JH, TM, RF-S, and MA: writing—review and editing. AC and PN: supervision, project administration, and funding acquisition. All authors have read and approved the final manuscript.

## Funding

This study is part of the research program of the General Foundation of the University of Salamanca, through the International Centre on Aging (CENIE), within the framework of the Programme for a Longevity Society (0551_PSL_6_E), a project co-financed by the European Regional Development Fund (ERDF) through the Interreg VA Spain-Portugal Programme (POCTEP) 2014–2020.

## Conflict of interest

The authors declare that the research was conducted in the absence of any commercial or financial relationships that could be construed as a potential conflict of interest.

## Publisher’s note

All claims expressed in this article are solely those of the authors and do not necessarily represent those of their affiliated organizations, or those of the publisher, the editors and the reviewers. Any product that may be evaluated in this article, or claim that may be made by its manufacturer, is not guaranteed or endorsed by the publisher.
